# The Markovian Shot-noise Risk Model: A Numerical Method for Gerber-Shiu Functions

**DOI:** 10.1007/s11009-023-10001-w

**Published:** 2023-02-09

**Authors:** Simon Pojer, Stefan Thonhauser

**Affiliations:** Institute of Statistics, Graz University of Technology, Kopernikusgasse 24, Graz, 8010 Austria

**Keywords:** Gerber-Shiu functions, Markov processes, Weak convergence, Shot-Noise, Risk theory, 60J25, 60J27, 91G05, 91G60

## Abstract

In this paper, we consider discounted penalty functions, also called Gerber-Shiu functions, in a Markovian shot-noise environment. At first, we exploit the underlying structure of piecewise-deterministic Markov processes (PDMPs) to show that these penalty functions solve certain partial integro-differential equations (PIDEs). Since these equations cannot be solved exactly, we develop a numerical scheme that allows us to determine an approximation of such functions. These numerical solutions can be identified with penalty functions of continuous-time Markov chains with finite state space. Finally, we show the convergence of the corresponding generators over suitable sets of functions to prove that these Markov chains converge weakly against the original PDMP. That gives us that the numerical approximations converge to the discounted penalty functions of the original Markovian shot-noise environment.

## Introduction and Overview

The introduction of the family of penalty functions by Gerber and Shiu in Gerber and Shiu (1998) had and still has a huge impact on the field of ruin theory. This unifying approach, generalizes previously considered risk measures and allows a comprehensive analysis of the ruin event of an insurance portfolio. Since then, Gerber-Shiu functions were analysed in different types of risk models. For example in the renewal model in Gerber and Shiu (2005), Li and Garrido (2005) and Willmot and Dickson (2003), the Markov modulated model in Zhang (2008), and the Björk-Grandell model in Schmidli (2010). The case of spectrally negative Lévy risk processes was already considered in Garrido and Morales (2006) and resolved in a very general form by the so-called quintuple law derived in Doney and Kyprianou (2006).

Initially, the main aim was to establish explicit formulas, which allow for direct calculation of discounted penalty functions. This was successfully done in the classical and renewal models, if the claim sizes are exponentially or phase-type distributed. Due to the increasing complexity of underlying models and considered penalty functions, this is generally hardly possible nowadays. Since simulation techniques like (quasi-)Monte Carlo methods are time-consuming and not always directly implementable, there is an increasing effort in finding efficient numerical procedures to determine suitable approximations of penalty functions for more complex models. Exemplary contributions are Chau et al. (2015), Diko and Usábel (2011), Lee et al. (2021), and Preischl et al. (2018). For the renewal risk model, Strini and Thonhauser (2020) introduced a numerical scheme based on a discretization of the corresponding generator to determine discounted penalty functions depending on a local cost functional and the deficit at ruin.

We consider Gerber-Shiu functions in the context of a Markovian shot-noise environment. The motivation for using the Markovian shot-noise model is the modelling of disasters, like earthquakes, as it was applied in Dassios and Jang (2003) in the context of pricing of reinsurance of catastrophic events. A generalized version of this model was considered by Albrecher and Asmussen (2006), who were interested in the asymptotic behaviour of the ruin probability in a general shot-noise model and derived exponentially decaying upper and lower bounds. Further extensions of this model are introduced by Stabile and Torrisi (2010), who considered heavy-tailed claim events, and Macci and Torrisi (2011), considering a non-constant premium rate. Recently, Pojer and Thonhauser (2022) were able to show the convergence behaviour of the ruin probability in the Markovian model.

In this contribution we are able to deal with Gerber-Shiu functions in their full generality. The introduction of an additional process, allows us to include functions depending on the surplus before ruin. By the underlying structure of piecewise-deterministic Markov processes, we can represent these discounted penalty functions as solutions to Feynman-Kac type partial integro-differential equations. Since there is no evidently explicit solution to the resulting equations, we develop a scheme to solve these equations numerically. First, we resolve the problem of the unboundedness of the involved intensity process. In a second step, we discretize the bounded version of the partial integro-differential equations and solve the corresponding system of linear equations. The obtained numerical solutions correspond to Gerber-Shiu functions of approximating Markov chains with finite state space. Eventually, we use weak convergence on the Skorokhod space of càdlàg functions to obtain a convergence result for the determined function values.

This paper is organized in the following way. In Section 2, we define the considered model, the concept of Gerber-Shiu functions, and their analytic properties. In Section 3, we introduce families of auxiliary processes used to approximate the original PDMPs of the Markovian shot-noise model and motivate the proposed numerical scheme. In Section 4, we show convergence of the numerical approximation by exploiting convergence in distribution of processes over the space of càdlàg functions. Finally, in Section 5, we give examples that show the performance of the proposed numerical scheme.

## Risk Model and Gerber-Shiu Functions

At first, we briefly introduce the considered Markovian shot-noise model as it is also used in Pojer and Thonhauser (2022). For this, we consider a probability space $\left( \Omega , \mathcal {F}, \mathbb {P} \right)$, which is assumed to be big enough to carry all of the subsequently defined stochastic objects.

### Definition 1

Let $\lambda$ and $\gamma$ be positive constants and $N^\rho$ a homogeneous Poisson process with intensity $\rho$ and jump times $\left\{ T_i^\rho \right\} _{i\mathop{\ge} 1}$. Let further $\left\{ Y_i \right\} _{i\mathop{\ge} 1}$ be i.i.d. copies of a positive random variable *Y* with distribution $F_Y$ independent of $N^\rho$. Then, the intensity process $\left\{ \lambda _t \right\} _{t \mathop{\ge} 0}$ given by

$$\begin{aligned} \lambda _t:= \lambda e^{-\gamma t} + \sum _{i\mathop{=}1}^{N^\rho _t} Y_i e^{-\gamma (t-T_i^\rho )}, \end{aligned}$$

is called Markovian shot-noise process.

Using this, we can define the surplus process in the following way.

### Definition 2

Let *N* be a Cox process whose stochastic intensity is a Markovian shot-noise process $\left\{ \lambda _t\right\} _{t \mathop{\ge} 0}$ and $\left\{ U_i \right\} _{i \mathop{\ge} 1}$ an i.i.d. sequence of positive random variables, independent of *N* and $\lambda$, with distribution function $F_U$. Let further *c* be a positive and *x* a non-negative constant. Then, the surplus process $\left\{ X_t \right\} _{t \mathop{\ge} 0}$ is given by

$$\begin{aligned} X_t= x +ct- \sum _{i\mathop{=}1}^{N_t} U_i. \end{aligned}$$

The Markovian shot-noise model was used by Dassios and Jang (2003) to model catastrophic events like earthquakes. A single catastrophic event, e.g. the earthquake, increases the intensity by a random quantity *Y*, called shock, and induces $Poi(Y/\gamma )$ many claims, called a cluster, which do not occur immediately, but instead they will be reported over a period of time. This allows us the following interpretation of the involved parameters and random variables. The parameter $\rho$ is the inverse of the expected time between two catastrophic events. Since the total number of claims due to a single catastrophe is $Poi(Y/\gamma )$, we have that the random variable $Y/\gamma$ determines the distribution of the number of claims in a single cluster. The decay parameter $\gamma$ determines how long it will take until all claims of the cluster are reported and paid. Despite the easy interpretation, it might be hard to estimate these quantities, especially the distribution of *Y*. 

From now on, we will assume the following:

### Assumption 1

Assume that the net profit condition $c> \frac{\rho }{\gamma }\mathbb {E}\left[ U\right] \mathbb {E}\left[ Y\right]$ holds, where *U* has distribution $F_U$ and is independent of all other stochastic objects introduced so far.

In many cases, the ruin probability itself is not a satisfying measure of the risk in the given model. One well-established much more general approach is to use Gerber-Shiu functions. For the precise setup, we follow the presentation used in Schmidli (2010). Let *w*(*x*, *y*) be a continuous and bounded function and $\kappa > 0$. Then, the corresponding Gerber-Shiu function is defined by

$$\begin{aligned} g_\kappa (x,\lambda ):= \mathbb {E}_{(x,\kern.10em\lambda )}\left[ e^{-\kappa \tau } w(X_{\tau -}, -X_{\tau }) I_{\left\{ \tau \mathop{<} \infty \right\} }\right] , \end{aligned}$$

for $(x,\lambda ) \in [0,\infty ) \times (0,\infty )$. Further, $g_\kappa (x,\lambda ) =0$ for $x< 0$ or $\lambda \le 0$. Here, the expectation $\mathbb {E}_{(x,\kern.10em\lambda )}$ is the expectation with initial conditions $X_0=x$ and $\lambda _0\mathop{=}\lambda$. Even though this representation is commonly used, it is not satisfying in our case. Since we want to exploit weak convergence of càdlàg processes to justify our numerical scheme, we have to extend the definition of GS-functions.

### Definition 3

Let $\left\{ \lambda _t\right\} _{t \mathop{\ge} 0}$ denote the Markovian shot-noise process and $\left\{ X_t\right\} _{t \mathop{\ge} 0}$ the corresponding surplus process. As a third process define $\left\{ m \right\} _{t \mathop{\ge} 0}$ as $m_t:= U_{N_t}$, the process which remembers the size of the latest claim. Using these processes we define for a continuous and bounded function *w*, and a constant $\kappa > 0$ the Gerber-Shiu function

$$\begin{aligned} g_\kappa (x,m,\lambda ) = \mathbb {E}_{(x,\kern.10em m,\kern.10em \lambda )} \left[ w\left( X_\tau +m_\tau , -X_\tau \right) e^{- \kappa \tau } I_{\left\{ \tau \mathop{<} \infty \right\} } \right] . \end{aligned}$$

As already mentioned before, the main advantage of this representation is, that we use the càdlàg process $m_t$ instead of the làdcàg process $X_{t-}$. Since *m* is a sequence of *i*.*i*.*d*. random variables, the value $m_\tau$ does not depend on the current level *m*, i.e. the size of the latest claim. Therefore, we will omit *m* in future and write still $g_\kappa (x,\lambda )$ for the GS-function. For the sake of completeness, we define the filtration $\left\{ \mathcal {F}_t \right\} _{t\,\ge\, 0}$ to be the natural filtration of the multivariate process $\left\{ (X_t, m_t, \lambda _t) \right\} _{t \mathop{\ge} 0}$. In this setting, the multivariate process is a strong Markov process with respect to this filtration.

To obtain a partial integro-differential equation which is satisfied by the GS-functions, we use the Markovian structure of our model. The process $\left\{ (X_t,m_t,\lambda _t) \right\} _{t\,\ge\, 0}$ is a piecewise-deterministic Markov process (PDMP) with generator

$$\begin{aligned} \mathcal {A}\,f(x,m,\lambda ) = c \frac{\partial f(x,m,\lambda )}{\partial x}&- \gamma \lambda \frac{\partial f(x,m,\lambda )}{\partial \lambda } + \lambda \int _0^\infty \! f(x-u,u,\lambda ) \, F_U(\mathrm{d}u) \\ {}&+ \rho \int _0^\infty \! f(x,m,\lambda + y) \, F_Y(\mathrm{d}y) - (\lambda + \rho )\, f(x,m,\lambda ), \end{aligned}$$

which is certainly well-defined for all bounded and continuously differentiable functions *f*. Since our Gerber-Shiu functions are generally not continuously differentiable, we use the general definition of the generator of a PDMP from Rolski et al. (1999). For a function *f*, the path-derivative is defined by

$$\begin{aligned} \delta _\phi \ f(x,m,\lambda ):= \lim _{h \mathop{\rightarrow} 0} \frac{f(x+ch, m, \lambda e^{- \gamma h})-f(x,m,\lambda )}{h}. \end{aligned}$$

Then, the domain of the generator of our PDMP consists of all functions *f*, which are path-differentiable a.e. and satisfy that for all $t \ge 0$,

$$\begin{aligned} \mathbb {E}_{(x,\kern.10em\lambda )} \left[ \sum _{i\mathop{=}1}^{N_t} \left|\, f(X_{T_i},m_{T_i},\lambda _{T_i}) \right.\right.&-\left.\left. f(X_{T_i-},m_{T_i-},\lambda _{T_i-}) \right| \right. \\ & +\left.\sum _{i\mathop{=}1}^{N^\rho _t} \left|\, f(X_{T^\rho _i},m_{T^{\lambda }_i},\lambda _{T^\rho _i}) - f(X_{T^\rho _i-},m_{T^{\rho }_i-},\lambda _{T^\rho _i-}) \right| \right] < \infty . \end{aligned}$$

For such a function *f*, the generator is characterized by

$$\begin{aligned} \mathcal {A}\,f(x,m,\lambda ) =\delta _\phi \ f(x,m,\lambda )&+ \lambda \int _0^\infty \! f(x-u,u,\lambda ) \, F_U(\mathrm{d}u) \\ {}&+ \rho \int _0^\infty \! f(x,m,\lambda + y) \, F_Y(\mathrm{d}y) - (\lambda + \rho )\, f(x,m,\lambda ). \end{aligned}$$

### Theorem 1

The Gerber-Shiu functions are in the domain of the generator of the PDMP $\left\{ (X_t,m_t,\lambda _t)\right\} _{t\,\ge\, 0}$.

### Proof

To show this, we prove that the Gerber-Shiu functions are path-differentiable and bounded.

For the path-differentiability, we follow the line of arguments as given in Strini and Thonhauser (2020). Define for some deterministic $r>0$ the bounded stopping time $\nu = r \wedge T_1$, where $T_1$ denotes the first jump-time of the PDMP. Doing this we get

$$\begin{aligned} g_\kappa (x,\lambda )&= \mathbb {E}_{(x,\kern.10em\lambda )}\left[ e^{-\kappa \tau } w(X_{\tau }+m_\tau , -X_{\tau }) I_{\left\{ \tau\, <\, \infty \right\} }\right] \\ {}&= \mathbb {E}_{(x,\kern.10em\lambda )}\left[ e^{-\kappa \nu } \mathbb {E}\left[ \left. e^{-\kappa (\tau \mathop{-}\nu )} w(X_{\tau }+m_\tau ,- X_{\tau }) I_{\left\{ \tau \mathop{<} \infty \right\} }\right| \mathcal {F}_{\nu } \right] \right] . \end{aligned}$$

Now, there are two cases. Either $\nu =r$ or $\nu =T_1$. Using this, we get

$$\begin{aligned}&g_\kappa (x,\lambda ) =: e^{-\int _0^r \! (\lambda e^{-\gamma s}\,+\,\rho ) \, \mathrm{d}s} e^{-\kappa r} g_\kappa (x+cr,\lambda e^{-\gamma r}) \ + \int _0^r \! H(s) \, \mathrm{d}s, \end{aligned}$$

where

$$\begin{aligned}H(s)&= (\lambda e^{-\gamma s }+ \rho )e^{- \int _0^s (\lambda e^{-\gamma u}\,+\,\rho ) \, \mathrm{d}u } e^{-\kappa s} \left( \frac{\rho }{\lambda e^{-\gamma s}+\rho } \int _0^\infty \! g_\kappa (x+cs,\lambda e^{-\gamma s}+y) \, F_Y(\mathrm{d}y) \right. \\ &\quad+\left. \frac{\lambda e^{-\gamma s}}{\lambda e^{-\gamma s} + \rho } \left( \int _0^{x\mathop{+}cs} \! g_\kappa (x\,+\,cs-u,\lambda e^{-\gamma s}) \, F_U( \mathrm{d}u)\right.\right.\\& \quad+\left.\left. \int _{x\,+\,cs}^\infty w(x+cs, u-x-cs) \, F_U(\mathrm{d}u) \right) \right) . \end{aligned}$$

Adding and subtracting $e^{-\int _0^r \! (\lambda e^{-\gamma s}\,+\,\rho \,+\,\kappa ) \, \mathrm{d}s} g_\kappa (x,\lambda )$ and rearranging gives us

$$\begin{aligned} \frac{g_\kappa (x+cr,\lambda e^{- \gamma r})-g_\kappa (x,\lambda )}{r}=&\ \ \frac{e^{\int _0^r (\lambda e^{-\gamma s} \mathop{+} \rho \mathop{+} \kappa ) \, \mathrm{d}s } -1}{r} g_\kappa (x,\lambda ) \\ {}&- \frac{e^{\int _0^r (\lambda e^{-\gamma s} \mathop{+} \rho \mathop{+} \kappa ) \, \mathrm{d}s }}{r} \int _0^r H(s) \, \mathrm{d}s. \end{aligned}$$

The integral over *H* is differentiable in $r=0$ from the right with derivative *H*(0). Hence, for $r \rightarrow 0$ we have

$$\begin{aligned} \lim _{r\, \rightarrow \,0}\frac{g_\kappa (x+cr,\lambda e^{- \gamma r})-g_\kappa (x,\lambda )}{r} =&\ \ (\lambda + \rho + \kappa ) g_\kappa (x,\lambda ) + H(0) \\=&\ \ (\lambda + \rho + \kappa ) g_\kappa (x,\lambda )- \rho \int _0^\infty g_\kappa (x,\lambda +y) \, F_Y(\mathrm{d}y) \\ {}&- \lambda \left( \int _0^x g_\kappa (x-u, \lambda ) \, F_U(\mathrm{d}u) + \int _x^\infty w(x,u-x) \, F_U( \mathrm{d}u) \right) , \end{aligned}$$

which gives us the differentiability of *g* along the paths of our PDMP. The integrability is an immediate consequence of the boundedness of *w*. $\square$

If we use the derived form of the path-derivative of $g_\kappa$ in the definition of the generator, we see that the GS-function solves the partial integro-differential equation

$$\begin{aligned} \mathcal {A}g_\kappa (x,\lambda ) = \kappa g_\kappa (x,\lambda ) -\lambda \int _x^\infty \left( w(x,u-x) - g_\kappa (x-u,\lambda )\right) \,F_U(\mathrm{d}u) , \end{aligned}$$

on $(x,\lambda ) \in [0,\infty ) \times (0, \infty )$. But, we still have to show that it is its unique solution.

### Theorem 2

Let $g_\kappa$ be a GS-function with some $\kappa > 0$. Then $g_\kappa$ is the unique bounded solution to the partial integro-differential equation (PIDE)

$$\begin{aligned} \delta _\phi \ f(x,\lambda )&+ \rho \int _0^\infty f (x, \lambda +y) \, F_Y(\mathrm{d}y) + \lambda \left( \int _0^x f(x-u,\lambda ) \, F_U(\mathrm{d}u) \right.\\&+\left. \int _x^\infty w(x,u-x) \, F_U(\mathrm{d}u)\right) -( \kappa +\lambda +\rho )\, f (x,\lambda ) =0, \end{aligned}$$

for $$(x,\lambda ) \in [0,\infty ) \times (0, \infty )$$.

### Proof

As already shown, the GS-function is bounded and solves the equation stated above. Now, observe that the PIDE can be rewritten in terms of the generator of the PDMP by

$$\begin{aligned} \mathcal {A}\,f(x,\lambda ) - \kappa f(x,\lambda ) - \lambda \int _x^\infty (\,f(x-u,\lambda )-w(x,u-x)) \, F_U(\mathrm{d}u) =0, \end{aligned}$$

for $(x,\lambda ) \in [0,\infty ) \times (0, \infty )$. Let $h: \mathbb {R}^2 \rightarrow \mathbb {R}$ be an arbitrary bounded solution of this equation, $\kappa > 0$, and *S* a bounded stopping time. Since *h* is path-differentiable and bounded, it is in the domain of the generator $\mathcal {A}$, which we use, to get

$$\begin{aligned}h(x,\lambda )&= \mathbb {E}_{(x,\kern.10em\lambda )}\left[ e^{-\kappa S} h(X_S,\lambda _S) - \int _0^S e^{-\kappa v} (\mathcal {A}\,h(X_v,\lambda _v) - \kappa h(X_v,\lambda _v) ) \mathrm{d}v \right] \\&= \mathbb {E}_{(x,\kern.10em\lambda )}\left[ e^{-\kappa S} h(X_S,\lambda _S) - \int _0^S e^{-\kappa v} \lambda _v \int _{X_{v}}^\infty (h(X_v-u,\lambda _v)-w(X_v,u-X_v)) \, F_U(\mathrm{d}u)\, \mathrm{d}v \right] . \end{aligned}$$

Using this representation for the bounded stopping time $\tau \wedge t$, yields

$$\begin{aligned} h(x,\lambda ) =&\ \mathbb {E}_{(x,\kern.10em\lambda )} \left[ e^{-\kappa \tau \mathop{\wedge} t}h(X_{\tau \mathop{\wedge} t}, \lambda _{\tau \mathop{\wedge} t}) \right] \\ {}&- \mathbb {E}_{(x,\kern.10em\lambda )}\left[ \int _0^{\tau\, \wedge\, t} e^{-\kappa v} \lambda _v \int _{X_v}^\infty h(X_v-u,\lambda _v) \, F_U(\mathrm{d}u) \,\mathrm{d}v \right] \\ {}&+\mathbb {E}_{(x,\kern.10em\lambda )}\left[ \int _0^{\tau\, \wedge\, t} e^{-\kappa v} \lambda _v \int _{X_v}^\infty w(X_v,u-X_v) \, F_U(\mathrm{d}u) \, \mathrm{d}v \right] . \end{aligned}$$

Let us now focus on the third term. Using that *N* is a counting process with intensity $\lambda$, we can rewrite this to

$$\begin{aligned}&\mathbb {E}_{(x,\kern.10em\lambda )}\left[ \int _0^{\tau\, \wedge\, t} e^{-\kappa v} \lambda _v \int _{X_v}^\infty w(X_v,u-X_v) \, F_U(\mathrm{d}u)\, \mathrm{d}v \right] \\&= \mathbb {E}_{(x,\kern.10em\lambda )}\left[ \int _{(0,\,\tau\, \wedge\, t]} e^{-\kappa v} \int _{X_{v-}}^\infty w(X_{v-},u-X_{v-}) \, F_U(\mathrm{d}u) \, \mathrm{d}N_v \right] \\ {}&= \mathbb {E}_{(x,\kern.10em\lambda )}\left[ \sum _{i\mathop{=}1}^{N_{\tau \mathop{\wedge} t}} e^{-\kappa T_i} \int _{X_{T_i-}}^\infty w(X_{T_i-},u-X_{T_i-}) \, F_U(\mathrm{d}u) \right] \\ &= \mathbb {E}_{(x,\kern.10em\lambda )}\left[ \sum _{i\mathop{=}1}^{N_{\tau \mathop{\wedge} t}} e^{-\kappa T_i} \mathbb {E}_{(x,\kern.10em\lambda )}\left[ w(X_{T_i-},U_i\mathop{-}x_{T_i-})I_{\left\{ U_i \,>\,X_{T_i-}\right\} } \, \left| \, \mathcal {F}_{T_i-} \, \right. \right] \right] . \end{aligned}$$

Since $I_{\left\{ U_i\, >\,X_{T_i-}\right\} }=0$ for all $T_i< \tau$ and 1 for $T_i=\tau$, the sum is 0 if $\tau >t$ and $e^{-\kappa \tau } \mathbb {E}_{(x,\kern.10em\lambda )}\left[ w(X_{\tau -}, -X_\tau )\, \left| \, \mathcal {F}_{\tau -} \,\right. \right]$ if $\tau \le t$. Consequently,

$$\begin{aligned}&\mathbb {E}_{(x,\kern.10em\lambda )}\left[ \sum _{i\mathop{=}1}^{N_{\tau \mathop{\wedge} t}} e^{-\kappa T_i} \mathbb {E}_{(x,\kern.10em\lambda )}\left[ w(X_{T_i-},U_i\mathop{-}x_{T_i-})I_{\left\{ U_i \mathop{>}X_{T_i-}\right\} } \, \left| \, \mathcal {F}_{T_i-} \, \right. \right] \right] \\&= \mathbb {E}_{(x,\kern.10em\lambda )}\left[ \sum _{i\mathop{=}1}^{N_{\tau \mathop{\wedge} t}} e^{-\kappa T_i} \mathbb {E}_{(x,\kern.10em\lambda )}\left[ w(X_{T_i-},U_i\mathop{-}x_{T_i-})I_{\left\{ T_i\mathop{=}\tau \right\} } \, \left| \, \mathcal {F}_{T_i-} \, \right. \right] \right] \\ &=\mathbb {E}_{(x,\kern.10em\lambda )}\left[ e^{-\kappa \tau } w(X_{\tau -},-X_\tau )I_{\left\{ \tau \mathop{\le} t \right\} } \right] . \end{aligned}$$

The same arguments yield

$$\begin{aligned} \mathbb {E}_{(x,\kern.10em\lambda )}\left[ \int _0^{\tau\, \wedge\, t} e^{-\kappa v} \lambda _v \int _{X_v}^\infty h(X_v-u,\lambda _v) \, F_U(\mathrm{d}u) \,\mathrm{d}v \right] = \mathbb {E}_{(x,\kern.10em\lambda )}\left[ e^{-\kappa \tau } h(X_{\tau },\lambda _\tau )I_{\left\{ \tau\, \le\, t \right\} } \right] . \end{aligned}$$

Hence,

$$\begin{aligned} h(x,\lambda ) =&\ \mathbb {E}_{(x,\kern.10em\lambda )}\left[ e^{-\kappa \tau \mathop{\wedge} t} h(X_{\tau \mathop{\wedge} t}, \lambda _{\tau \mathop{\wedge} t}) \right] \\&- \mathbb {E}_{(x,\kern.10em\lambda )}\left[ e^{-\kappa \tau } h(X_{\tau },\lambda _\tau )I_{\left\{ \tau \mathop{\le} t \right\} } \right] \\ {}&+ \mathbb {E}_{(x,\kern.10em\lambda )}\left[ e^{-\kappa \tau } w(X_{\tau -},-X_\tau )I_{\left\{ \tau \mathop{\le} t \right\} } \right] \\=&\ \ \mathbb {E}_{(x,\kern.10em\lambda )}\left[ e^{-\kappa t} h(X_{ t}, \lambda _{ t}) I_{\left\{ \tau \mathop{>}t \right\} }\right] \\&+ \mathbb {E}_{(x,\kern.10em\lambda )}\left[ e^{-\kappa \tau } w(X_{\tau -},-X_\tau )I_{\left\{ \tau \mathop{\le} t \right\} } \right] . \end{aligned}$$

Since *h* is bounded, we can find a positive constant *K* such that

$$\begin{aligned} \left| \mathbb {E}_{(x,\kern.10em\lambda )}\left[ e^{-\kappa t} h(X_{ t}, \lambda _{ t})I_{\left\{ \tau >t \right\} } \right] \right| \le Ke^{-\kappa t}. \end{aligned}$$

Using this, we finally get that

$$\begin{aligned} h(x,\lambda )&= \lim _{t \rightarrow \infty } \mathbb {E}_{(x,\kern.10em\lambda )}\left[ e^{-\kappa t} h(X_{ t}, \lambda _{ t}) I_{\left\{ \tau >t \right\} }\right] + \mathbb {E}_{(x,\kern.10em\lambda )}\left[ e^{-\kappa \tau } w(X_{\tau -},-X_\tau )I_{\left\{ \tau \le t \right\} } \right] \\ {}&=\lim _{t \rightarrow \infty }\mathbb {E}_{(x,\kern.10em\lambda )}\left[ e^{-\kappa \tau } w(X_{\tau -},-X_\tau )I_{\left\{ \tau \le t \right\} } \right] \\ {}&= \mathbb {E}_{(x,\kern.10em\lambda )}\left[ e^{-\kappa \tau } w(X_{\tau -},-X_\tau )I_{\left\{ \tau <\infty \right\} } \right] . \end{aligned}$$

$$\square$$

## Auxiliary Processes

As already shown in the previous section, the function $g_\kappa$ satisfies a partial integro-differential equation (PIDE). Generally, this equation cannot be solved explicitly. Hence, we need some numerical scheme that allows us to calculate an approximation of the desired value. An intuitive way to do so, is to bound the state space and suitably discretize the PIDE on the bounded domain. This results in a system of linear equations which we can solve. Our approach is to approximate a bounded version of the PDMP by Markov chains, determine the corresponding Gerber-Shiu functions and show that these converge to the original ones.

### Bounded Processes

The first step of the approximation procedure is to bound some components of the processes in a suitable way.

#### Definition 4

Let $b>0$, $\lambda _{max}(b)>0$, $U_{max}(b)>0$ and $Y_{max}(b)>0$ such that $\lim _{b \mathop{\rightarrow} \infty } \lambda _{max}(b) = \lim _{b \mathop{\rightarrow} \infty } U_{max}(b) = \lim _{b \mathop{\rightarrow} \infty } Y_{max}(b) = \infty$. Then, we define the bounded intensity process by

$$\begin{aligned} \lambda ^{(b)}_t = \lambda e^{-\gamma t} + \sum _{i\mathop{=}1}^{N^\rho _t} Y^{(b)}_i e^{-\gamma (t\mathop{-}T^\rho _i)}. \end{aligned}$$

Here, the distribution of the random variable $Y_j^{(b)}$ depends on the original *j*-th shock and the pre-jump location of the process $\lambda ^{(b)}$ in the following way: define the random variable $\bar{Y}_j = Y_j I_{\left\{ Y_j \mathop{\le} Y_{max}(b) \right\} } + Y_{max}(b) I_{\left\{ Y_j \mathop{>}Y_{max}(b) \right\} }$, then the bounded shocks are given by

$$\begin{aligned} Y_j^{(b)} = \bar{Y}_j I_{\left\{ \lambda ^{(b)}_{T^\rho _j-} + \bar{Y}_j \mathop{\le} \lambda _{max}(b)\right\} } + (\lambda _{max}(b) - \lambda ^{(b)}_{T^\rho _j-}) I_{\left\{ \lambda ^{(b)}_{T^\rho _j-} + \bar{Y}_j \mathop{>} \lambda _{max}(b)\right\} }. \end{aligned}$$

This might seem complicated, but it ensures, that our new shocks have bounded support and the whole process $\lambda ^{(b)}$ does not leave the bounded state space $(0, \lambda _{max}(b)]$. Given this new process, we define our new counting process $N^{(b)}$ using the acceptance-rejection method, also called thinning. Let *T* be a jump time of the original counting process *N* and $U\sim U[0,1]$. We accept the jump time for the new jump process if $\frac{\lambda ^{(b)}_T}{\lambda _T} \ge U$. That gives us that $N^{(b)}$ is a Cox process with intensity $\lambda ^{(b)}$, whose jump times coincide with jump times of our original process. Defining the sequence of bounded claims by $U_j^{(b)} = U_j I_{\left\{ U_j\, \le\, U_{max}(b) \right\} } + U_{max}(b) I_{\left\{ U_j\, >\,U_{max}(b) \right\} }$, we can further define

$$\begin{aligned} X^{(b)}_t = x + ct - \sum _{i\mathop{=}1}^{N^{(b)}_t} U^{(b)}_i, \end{aligned}$$

and $$m_t^{(b)} = U^{(b)}_{N^{(b)}_t}$$.

Even though, $\left\{ Y^{(b)}_i\right\} _{i\, \in\, \mathbb {N}}$ is no longer an i.i.d. sequence, this new triplet of processes is again a PDMP with generator

$$\begin{aligned} \mathcal {A}^{(b)} f(x,m,\lambda )=&\ \ \delta _\phi \ f(x,m,\lambda ) + \lambda \int _0^{U_{max}(b)} f(x-u,u,\lambda ) \, F_U(\mathrm{d}u) \\ {}&+ \lambda \ f(x-U_{max}(b),U_{max}(b), \lambda ) \,\mathbb {P}\left[ U>U_{max}(b)\right] \\ {}&+ \rho \int _0^{Y_{max}(b)} f(x, m,\min \left\{ \lambda _{max}(b), \lambda +y \right\} ) \, F_Y(\mathrm{d}y) \\ {}&+ \rho \, f(x,m,\min \left\{ \lambda _{max}(b), \lambda +Y_{max}(b) \right\} )\mathbb {P}\left[ Y> Y_{max}(b)\right] \\ {}&- (\lambda +\rho )\,f(x,m,\lambda ), \end{aligned}$$

or alternatively we write for convenience

$$\begin{aligned} \mathcal {A}^{(b)} f(x,m,\lambda )=&\ \ \delta _\phi \ f(x,m,\lambda ) + \lambda \int _{(0,\kern 0.10em U_{max}(b)]} f(x-u,u,\lambda ) \, F_{U^{(b)}}(\mathrm{d}u) \\ {}&+ \rho \int _{(0,\kern 0.10em Y_{max}(b)]} f(x, m, \lambda +y ) \, F_{Y^{(b)}}(\mathrm{d}y, \lambda ) - (\lambda +\rho )\,f(x,m,\lambda ). \end{aligned}$$

Having this, we can now define the GS-function of the bounded process.

#### Definition 5

Let $g_\kappa (x,\lambda )= \mathbb {E}_{(x,\kern.10em\lambda )} \left[ w(X_\tau +m_\tau , -X_\tau ) e^{-\kappa \tau } I_{\left\{ \tau \mathop{<} \infty \right\} } \right]$ be an arbitrary Gerber-Shiu function. Then, we define the corresponding GS-function of the bounded process by

$$\begin{aligned} g^{(b)}_\kappa (x,\lambda ) = \mathbb {E}_{(x,\kern.10em\lambda )} \left[ w(X^{(b)}_{\tau ^{(b)}}+m^{(b)}_{\tau ^{(b)}}, -X^{(b)}_{\tau ^{(b)}} ) e^{-\kappa {\tau ^{(b)}}} I_{\left\{ {\tau ^{(b)}} < \infty \right\} }\right] , \end{aligned}$$

where $$\tau ^ {(b)}= \inf \left\{ t \ge 0 \, \left| X^{(b)}_t \le 0 \, \right. \right\} .$$

We call these processes bounded, since the intensity process $\left\{ \lambda _t^{(b)} \right\} _{t \mathop{\ge} 0}$ and the random events $Y^{(b)}$ and $U^{(b)}$ are a.s. bounded. Despite this denomination, the multivariate process $\left\{ (X_t^{(b)}, m_t^{(b)},\lambda _t^{(b)}) \right\} _{t \mathop{\ge} 0}$ is not bounded. The surplus process $\left\{ X_t^{(b)} \right\} _{t \mathop{\ge} 0}$ is left unbounded since any change there would disturb the strictly monotone increasing drift. We could resolve this problem, by using external states, which could be introduced to change the deterministic flow. This would create an active boundary, i.e. an area where jumps occur deterministically. Unfortunately, this causes several problems in the proofs of weak convergence in Section 4.

### Discrete State Processes

Let us now fix some *b*, and $h>0$. We set $N_U = \left\lfloor \frac{U_{max}(b)}{h}\right\rfloor$ and $N_\lambda$ such that $\lim _{h \mathop{\rightarrow} 0} N_\lambda h = \infty$. Then we introduce a continuous-time Markov chain with countable state space approximating the bounded process the following way:

#### Definition 6

Define the state space

$$\left\{ (x_i,x_l,\lambda _j)\, \left| \, i, l \in \mathbb {Z}, \, 1 \le j \le N_\lambda \right. \right\} ,$$

where $x_i \mathop{=} cih$, and $\lambda _j:= \lambda _{max}(b) \exp \left( - \gamma (N_\lambda -j) h \right)$, and the probabilities $p^U_k = \mathbb {P}\left[ x_{k\mathop{-}1}< U^{(b)} \le x_k \right]$ for $k < N_U$ and $p^U_{N_U} = 1- \sum _{k\mathop{=}1}^{N_U-1} p^U_k$.

Further, we set for some fixed $\lambda _j$

$$N_Y(j) = \# \left\{ \lambda _{j\mathop{+}k} \, \left| \, k\ge 1, \, \lambda _{j\mathop{+}k}-\lambda _j \le Y_{max}(b) \right. \right\} ,$$

the number of points which we can reach from $\lambda _j$ with a bounded shock event. The corresponding probabilities are

$$p^Y_k(j) = \mathbb {P}\left[ \lambda _{j\mathop{+}k\mathop{-}1}-\lambda _j< Y^{(b)} \le \lambda _{j\mathop{+}k}-\lambda _j \right] \text { for }k < N_Y(j),$$

and $$p^Y_{N^Y(j)} (j) := 1 - \sum _{k\mathop{=}1}^{N_Y(j)\mathop{-}1} p^Y_k(j)$$.

Using this, we define the Markov chain on the discrete state space via its generator

$$\begin{aligned} \mathcal {A}^{(h,\kern.10em b)} f(x_i,x_l,\lambda _j) =&\ \frac{f(x_{i\mathop{+}1},x_l,\lambda _{j\mathop{-}1})-f(x_i,x_l,\lambda _j)}{h} \\ {}&+ \lambda _j \sum _{k\mathop{=}1}^{N_U} f(x_i\mathop{-}x_k, x_k, \lambda _j)\, p^U_k \\ {}&+ \rho \sum _{k\mathop{=}1}^{N_Y(j)} f(x_i,x_l, \lambda _{j\mathop{+}k}) \,p^Y_k(j)- (\lambda _j\mathop{+}\rho )\, f(x_i,x_l,\lambda _j), \end{aligned}$$

where we set $\lambda _{1\mathop{-}1} = \lambda _1$ and $\lambda _n = \lambda _{max}(b)$ for all $n \ge N_\lambda$.

This generator consists still of infinitely many expressions, due to the unbounded state-space. To bypass this, we have to introduce a third family of processes with finite state-space.

#### Definition 7

Let $\bar{x}$ be a positive constant and define $N_x = \left\lfloor \frac{\bar{x}}{ch}\right\rfloor$. Then, we define the finite state space by

$$\begin{aligned} \left\{ (x_i,x_l,\lambda _j) \, \left| \, -N_x \le i,l \le N_x, \, j \le N_\lambda \right. \right\} , \end{aligned}$$

where $x_i$ and $\lambda _j$ are as in the countable case. On this grid, we define the Markov chain $(X^{(\bar{x}, h, b)}, m^{(\bar{x}, h,b)}, \lambda ^{(\bar{x}, h, b)})$ by its generator. For $i<N_x$ and $j>1$ set

$$\begin{aligned} \mathcal {A}^{(\bar{x},h,b)} f(x_i,x_l,\lambda _j) =&\ \frac{f(x_{i\mathop{+}1},x_l,\lambda _{j\mathop{-}1})-f(x_i,x_l,\lambda _j)}{h} \\ {}&+ \lambda _j \sum _{k\mathop{=}1}^{\min \left( N_U,\kern.10em N_x\mathop{+}i\right) } f(x_{i\mathop{-}k}, x_k, \lambda _j)\, p^U_k \\ {}&+ \lambda _1 f(x_{-N_x}, \lambda _j)\, \left( 1- \sum _{k\mathop{=}1}^{\min \left( N_U, N_x\mathop{+}i\right) } p^U_k \right) \\ {}&+ \rho \sum _{k\mathop{=}1}^{N_Y(j)} f(x_i,x_l, \lambda _{j\mathop{+}k}) \,p^Y_k(j)- (\lambda _j\mathop{+}\rho )\, f(x_i,x_l,\lambda _j). \end{aligned}$$

For $i=N_x$ and $j>1$ set

$$\begin{aligned} \mathcal {A}^{(\bar{x},h,b)} f(x_{N_x},x_l,\lambda _j) =&\ \frac{f(x_{N_x},x_l,\lambda _{j\mathop{-}1})-f(x_{N_x},x_l,\lambda _j)}{h} \\ {}&+ \lambda _j \sum _{k\mathop{=}1}^{\min \left( N_U, 2N_x\right) } f(x_{N_x\mathop{-}k}, x_k, \lambda _j)\, p^U_k \\ {}&+ \lambda _j f(x_{-N_x}, \lambda _j) \,\left( 1- \sum _{k\mathop{=}1}^{\min \left( N_U, 2N_x\right) } p^U_k \right) \\ {}&+ \rho \sum _{k\mathop{=}1}^{N_Y(j)} f(x_{N_x},x_l, \lambda _{j\mathop{+}k}) \,p^Y_k(j)- (\lambda _j\mathop{+}\rho )\, f(x_{N_x},x_l,\lambda _j). \end{aligned}$$

For $i=N_x$ and $j=1$ set

$$\begin{aligned} \mathcal {A}^{(\bar{x},h,b)} f(x_{N_x},x_l,\lambda _1) =&\ \lambda _1 \sum _{k\mathop{=}1}^{\min \left( N_U, 2N_x\right) } f(x_{N_x\mathop{-}k}, x_k, \lambda _1)\, p^U_k \\ {}&+ \lambda _1 f(x_{-N_x}, \lambda _1) \,\left( 1- \sum _{k\mathop{=}1}^{\min \left( N_U, 2N_x\right) } p^U_k \right) \\ {}&+ \rho \sum _{k\mathop{=}1}^{N_Y(j)} f(x_{N_x},x_l, \lambda _{1\mathop{+}k}) \,p^Y_k(1)- (\lambda _1+\rho )\, f(x_{N_x},x_l,\lambda _1). \end{aligned}$$

Here, we write again $\lambda _{1\mathop{-}1}=\lambda _1$ and $\lambda _n = \lambda _{max}(b)$ for all $n \ge N_\lambda$.

Now, we can introduce the corresponding GS-function of the Markov chain with finite state space.

#### Lemma 3

Let $g_\kappa (x,\lambda )=\mathbb {E}_{(x,\kern.10em\lambda )}\left[ e^{- \kappa \tau } w(X_\tau + m_\tau , -X_\tau ) I_{\left\{ \tau <\infty \right\} }\right]$ be an arbitrary discounted penalty function of our original model. Then, we define the corresponding GS-function of the Markov chain with finite state space by

$$\begin{aligned} g^{(\bar{x}, h, b)}_\kappa (x_i,\lambda _j) = \mathbb {E}_{(x_i,\lambda _j)}\left[ e^{- \kappa \tilde{\tau }} w(X^{(\bar{x}, h, b)}_{\tilde{\tau }} + m^{(\bar{x}, h,b)}_{\tilde{\tau }}, -X^{(\bar{x}, h, b)}_{\tilde{\tau }})I_{ \left\{ \tilde{\tau }< \infty \right\} }\right] , \end{aligned}$$

where $\tilde{\tau }= \inf \left\{ t \ge 0 \, \left| \, X^{(\bar{x}, h, b)}_t \le 0 \right. \right\} .$ This function $g_\kappa ^{(\bar{x},h,b)}(x_i,\lambda _j)$ is the unique solution of the following finite system of linear equations:

$$\begin{aligned}\frac{f(x_{i\mathop{+}1},\lambda _{j\mathop{-}1})-f(x_i,\lambda _j)}{h}&- (\lambda _j\mathop{+}\rho +\kappa )\, f(x_i,\lambda _j) + \lambda _j \sum _{k\mathop{=}1}^{\min (i\,-\,1,N_U)} f(x_i\mathop{-}x_k, \lambda _j)\, p^U_k \\ {}&+ \lambda _j I_{\left\{ i\, \le\, N_U \right\} } \sum _{k\mathop{=}i}^{N_U} w(x_i, x_k-x_i)\, p^U_k \\&+ \rho \sum _{k\mathop{=}1}^{N_Y(j)} f(x_i, \lambda _{j\mathop{+}k}) \,p^Y_k(j)=0 \text { for } i < N_x,\,j > 1, \end{aligned}$$

$$\begin{aligned}\frac{f(x_{N_x},\lambda _{j\mathop{-}1})-f(x_{N_x},\lambda _j)}{h} &+ \lambda _j \sum _{k\mathop{=}1}^{N_U} f(x_{N_x}-x_k, \lambda _j)\, p^U_k \\ {}&+ \rho \sum _{k\mathop{=}1}^{N_Y(j)} f(x_{N_x}, \lambda _{j\mathop{+}k}) \,p^Y_k(j)\\&- (\lambda _j\mathop{+}\rho +\kappa )\, f(x_{N_x},\lambda _j)=0 \text { for } j>1, \end{aligned}$$

$$\begin{aligned}\frac{f(x_{i\mathop{+}1},\lambda _{1})-f(x_i,\lambda _1)}{h}&+ \lambda _1 \sum _{k\mathop{=}1}^{\min (i\,-\,1,N_U)} f(x_i\mathop{-}x_k, \lambda _1)\, p^U_k + \lambda _1 I_{\left\{ i \,\le\, N_U \right\} } \sum _{k\,=\,i}^{N_U} w(x_i, x_k-x_i)\, p^U_k \\ &+ \rho \sum _{k\mathop{=}1}^{N_Y(1)} f(x_i, \lambda _{1+k}) \,p^Y_k(1)- (\lambda _1\,+\,\rho\, +\,\kappa )\, f(x_i,\lambda _1)=0\, \text { for } i < N_x, \end{aligned}$$

and

$$\begin{aligned} \lambda _1 \sum _{k\mathop{=}1}^{N_U} f(x_{N_x}-x_k, \lambda _1)\, p^U_k + \rho \sum _{k\mathop{=}1}^{N_Y(1)} f(x_{N_x}, \lambda _{1+k}) \,p^Y_k(1)- (\lambda _1\,+\,\rho\, +\,\kappa )\, f(x_{N_x},\lambda _1)=0 . \end{aligned}$$

#### Proof

Since $\kappa >0$, the matrix corresponding to the above system of equations is strict diagonally dominant, hence regular. Since the GS-function solves the system, it is the unique solution. $\square$

## Convergence of Gerber-Shiu Functions

In this section, we will prove that our numerical scheme converges as $h \rightarrow 0$ and $b \rightarrow \infty$. For this, we want to exploit the convergence in distribution of processes as random variables on the Skorokhod space of càdlàg functions. This convergence implies the convergence of Skorokhod-continuous and bounded functionals of the corresponding processes. For further details on this metric space see (Ethier and Kurtz 2009, Chapter 3).

Since our processes are Markov processes, the main idea is to reduce this to the convergence of the corresponding generators. For Feller processes, these properties are equivalent as shown in (Kallenberg 2002, Theorem 19.25). Since our processes are not Feller, we will use Theorem 8.2 in Chapter 5 of Ethier and Kurtz (2009) to show the same. Consequently, we have to find a suitable subdomain of our generators such that the induced semigroup is strongly continuous on this set of functions. If this domain is convergence determining, e.g. if it contains $C^\infty _c$, and the generators converge for all *f* from this domain, then the corresponding processes converge weakly.

### Convergence of the Bounded Processes

#### Lemma 4

The generator $\mathcal {A}$ of the original PDMP generates a strongly continuous contraction semigroup $\left\{ T_t \right\} _{t \mathop{\ge} 0}$ on the $\Vert \cdot \Vert _{\infty }$-closure of the set

$$\begin{aligned} \mathcal {D}= \left\{ f \in C_b \, \left| \, \delta _\phi \ f \text { is path-continuous and } \mathcal {A}\,f \in C_b \right. \right\} , \end{aligned}$$

by $$T_t\, f(x,m,\lambda ) := \mathbb {E}_{(x,\kern.10em m,\kern.10em \lambda )} \left[\, f(X_t, m_t, \lambda _t) \right] .$$  

#### Proof

Since $\mathcal {A}$ is the generator of the Markov process $\left\{ (X_t, m_t, \lambda _t)\right\} _{t \mathop{\ge} 0}$, we have to show that $T_t$ maps this set into itself and is strongly continuous there.

By a small modification of the proof of Theorem 27.6 in Davis (1993), we can relax the needed assumption that the intensity is bounded. This gives us that for all bounded and continuous *f*, we have that $T_t\, f \in C_b$ too. By Theorem 7.7.4 of (Jacobsen 2006, pp. 181-182), the operators map path-differentiable functions satisfying $\Vert \mathcal {A}\,f\Vert _{\infty }$ into itself and satisfy $\mathcal {A}T_t\,f = T_t \mathcal {A}\,f$. By this, we get for all $f \in \mathcal {D}$ that $\mathcal {A}T_t \,f = T_t \mathcal {A}\,f \in C_b$.

The strong continuity is an immediate consequence of the boundedness of $\mathcal {A}f$. Consider $\left| T_t\, f(x,m,\lambda ) - f(x,m,\lambda )\right|$ for some fixed *t*. Then, it holds that

$$\begin{aligned} \left| T_t\, f(x,m,\lambda ) - f(x,m,\lambda )\right| = \left| \int _0^t \mathbb {E}_{(x,\kern.10em m,\kern.10em \lambda )}\left[ \mathcal {A}\,f(X_s,m_s,\lambda _s) \right] \, \mathrm{d}s \right| \le t \Vert \mathcal {A}\,f \Vert _{\infty }. \end{aligned}$$

Since this upper bound is independent of $(x,m,\lambda )$, we can let *t* tend to 0, which gives us that the contraction semigroup $T_t$ is strongly continuous in $t=0$. $\square$

#### Theorem 5

Let $f \in \mathcal {D}$ be arbitrary and $g= \mathcal {A}\,f$. Then, for all $k \ge 0$, $0 \le t_1< t_2< \cdots<t_k \le t < t+s$ and $h_1, \cdots , h_k \in C_b$ we have that

$$\begin{aligned} \lim _{b \mathop{\rightarrow} \infty } \mathbb {E}_{(x,\kern.10em m,\kern.10em \lambda )} \left[ \left(\, f(X^{(b)}_{t\mathop{+}s}, m^{(b)}_{t\mathop{+}s}, \lambda ^{(b)}_{t\mathop{+}s})\right. \right.&- \left. \left. f(X^{(b)}_{t}, m^{(b)}_{t}, \lambda ^{(b)}_{t})-\int _t^{t\mathop{+}s} g(X^{(b)}_{v}, m^{(b)}_{v}, \lambda ^{(b)}_{v})\, \mathrm{d}v\right) \right. \\ {}&\times\left. \prod _{i\mathop{=}1}^k h_i(X^{(b)}_{t_i}, m^{(b)}_{t_i}, \lambda ^{(b)}_{t_i}) \right] =0. \end{aligned}$$

#### Proof

For convenience, we will write $Z_t:= (X_t, m_t, \lambda _t)$, $Z^{(b)}_t := (X^{(b)}_t, m^{(b)}_t, \lambda ^{(b)}_t)$ and $z=(x,m,\lambda )$. At first, we will cover the case $k=0$. Let $f \in \mathcal {D}$ arbitrary and $g= \mathcal {A}\,f$. It is easy to see, that for every $b>0$, *f* is in the domain of the generator $\mathcal {A}^{(b)}$ too. Writing $g^{(b)}$ for $\mathcal {A}^{(b)}f$ we have that

$$\begin{aligned} \mathbb {E}_z \left[\, f(Z^{(b)}_{t\mathop{+}s}) - f(Z^{(b)}_{t})- \int _t^{t\mathop{+}s} g(Z^{(b)}_v) \, \mathrm{d}v \right] =&\ \mathbb {E}_z \left[\, f(Z^{(b)}_{t\mathop{+}s}) - f(Z^{(b)}_{t})- \int _t^{t\mathop{+}s} g^{(b)}(Z^{(b)}_v) \, \mathrm{d}v \right] \\ {}&+ \mathbb {E}_z \left[ \int _t^{t\mathop{+}s} g^{(b)}(Z^{(b)}_v) - g(Z^{(b)}_v) \, \mathrm{d}v \right] . \end{aligned}$$

The first term is the expectation of a zero mean martingale, hence 0. For the second term, we take a closer look at the difference of the generators $\mathcal {A}$ and $\mathcal {A}^{(b)}$ applied to the same function *f* in the same point $(x,m,\lambda )$:

$$\begin{aligned} \mathcal {A}\,f(x,m,\lambda ) =&\ \delta _\phi \ f(x,m,\lambda ) + \lambda \int _0^\infty f(x-u, u, \lambda ) \, F_U(\mathrm{d}u) \\ {}&+ \rho \int _0^\infty f(x,m,\lambda +y) \, F_Y(\mathrm{d}y) - (\lambda + \rho )\, f(x,m,\lambda ) \end{aligned}$$

and

$$\begin{aligned} \mathcal {A}^{(b)}f(x,m,\lambda ) =&\ \delta _\phi \ f(x,m,\lambda ) + \lambda \int _0^{U_{max}(b)} f(x-u, u, \lambda ) \, F_U(\mathrm{d}u) \\ {}&+ \rho \int _0^{Y_{max}(b)} f(x,m,\min \left\{ \lambda _{max}(b),\lambda +y\right\} ) \, F_Y(\mathrm{d}y) \\ {}&+ \lambda \, \mathbb {P}\left[ U> U_{max}(b) \right]\, f(x-U_{max}(b), U_{max}(b), \lambda ) \\ {}&+ \rho \, \mathbb {P}\left[ Y> Y_{max}(b) \right]\, f(x,m, \min \left\{ \lambda _{max}(b), \lambda + Y_{max}(b)\right\} ) \\ {}&- (\lambda + \rho )\, f(x,m,\lambda ) \end{aligned}$$

The derivatives coincide and so do the integrals from 0 to $U_{max}(b)$ and 0 to $\min \left\{ \lambda _{max}(b) - \lambda , Y_{max}(b)\right\}$ respectively. The absolute value of the remaining parts can be bounded by

$$\begin{aligned}&\left| \lambda \int _{U_{max}(b)}^\infty f(x-u,u,\lambda ) \, F_U(\mathrm{d}u) - \lambda \, \mathbb {P}\left[ U> U_{max}(b) \right]\, f(x-U_{max}(b), U_{max}(b), \lambda )\right| \\ {}&\quad+ \left| \rho \int _{\min \left\{ \lambda ^{(b)}_{max}-\lambda , Y_{max}(b) \right\} }^\infty f(x,m,\lambda +y) - \rho \,\mathbb {P}\left[ Y>\min \left\{ \lambda ^{(b)}_{max}-\lambda , Y_{max}(b) \right\} \right]\, f(x,m,\lambda _{max}^{(b)}) \right| \\ {}&\le 2 \lambda \Vert f\Vert _{\infty } \mathbb {P}\left[ U> U_{max}(b) \right] + 2 \rho \Vert f\Vert _{\infty }\, \mathbb {P}\left[ Y> \min \left\{ \lambda _{max}^{(b)}-\lambda , Y_{max}(b) \right\} \right] . \end{aligned}$$

This upper bound tends to 0 as $b \rightarrow \infty$ since $U_{max}(b)$, $\lambda _{max}(b)$ and $Y_{max}(b)$ tend to infinity but not uniformly in $\lambda$.

If we now get back to our expectation we see that

$$\begin{aligned} \left| \mathbb {E}_z \left[\, f(Z^{(b)}_{t\mathop{+}s}) - f(Z^{(b)}_{t}) \right. \right.&-\left. \left. \int _t^{t\mathop{+}s} g(Z^{(b)}_v) \, \mathrm{d}v \right] \right| \le \mathbb {E}_z \left[ \int _t^{t\mathop{+}s} \left| g^{(b)}(Z^{(b)}_v) - g(Z^{(b)}_v) \right| \, \mathrm{d}v \right] \\ \le&\ 2 \Vert f\Vert _{\infty } \rho \,\int _t^{t\mathop{+}s}\! \mathbb {P}_\lambda \left[ Y> \min \left\{ \lambda _{max}(b)-\lambda ^{(b)}_v ,Y_{max}(b) \right\} \right] \, \mathrm{d}v \\ {}&+ 2 \Vert f\Vert _{\infty } \int _t^{t\mathop{+}s}\! \mathbb {E}_\lambda \left[ \lambda ^{(b)}_v\right] \mathbb {P}\left[ U>U_{max}(b) \right] \, \mathrm{d}v \\ \le&\ 2 \Vert f\Vert _{\infty } \rho \, \int _t^{t\mathop{+}s} \!\mathbb {P}_\lambda \left[ Y> \min \left\{ \lambda _{max}(b)-\lambda ^{(b)}_v , Y_{max}(b) \right\} \right] \, \mathrm{d}v \\ &+ 2 \Vert f\Vert _{\infty } \int _t^{t\mathop{+}s} \! \mathbb {E}_\lambda \left[ \lambda _v\right] \mathbb {P}\left[ U>U_{max}(b) \right] \, \mathrm{d}v \\ \le&\ 2 \Vert f\Vert _{\infty }\rho \, \int _{t}^{t\mathop{+}s}\! \mathbb {P}_\lambda \left[ Y> \min \left\{ \lambda _{max}(b)-\lambda _v, Y_{max}(b) \right\} \right] \, \mathrm{d}v \\ {}&+ 2 \Vert f\Vert _{\infty } \left( \lambda + \frac{\rho }{\gamma }\mathbb {E}\left[ Y\right] \right) \mathbb {P}\left[ U>U_{max}(b) \right] . \end{aligned}$$

The second part tends to 0 as $b \rightarrow \infty$ but the first part still needs some work, since it depends on $\lambda _v$. For this, we remember that, given $\lambda _0 = \lambda$, $\lambda _v-\lambda$ with $v \le s+t$ can be bounded from above by the compound Poisson distributed random variable $\sum _{i\mathop{=}1}^{N^\rho _{t\mathop{+}s}}Y_i$. By this we get that

$$\mathbb {P}_\lambda \left[ Y> \min \left\{ \lambda _{max}(b)-\lambda _v, Y_{max}(b) \right\} \right] \le \mathbb {P}\,\left[ \lambda +Y+\sum _{i\mathop{=}1}^{N^\rho _{t\mathop{+}s}} Y_i> \min \left\{ \lambda _{max}(b), Y_{max}(b) \right\} \right] ,$$

which is independent of *v* and tends to 0 as *b* tends to infinity. By this, we have that

$$\begin{aligned} \lim _{b \mathop{\rightarrow} \infty }& \left| \mathbb {E}_z\left[ \int _t^{t\mathop{+}s} g^{(b)} (Z^{(b)}_v) - g(Z^{(b)}_v) \, \mathrm{d}v \right] \right| \\\le&\ \lim _{b \mathop{\rightarrow} \infty } 2\Vert f\Vert _{\infty } \left( \lambda + \frac{\rho }{\gamma }\mathbb {E}\left[ Y\right] \right) \mathbb {P}\,\left[ U>U_{max}(b) \right] \\&+\lim _{b \mathop{\rightarrow} \infty } s\rho \, \mathbb {P}\left[ \lambda +Y+\sum _{i\mathop{=}1}^{N^\rho _{t\mathop{+}s}} Y_i> \min \left\{ \lambda _{max}(b), Y_{max}(b) \right\} \right] =0. \end{aligned}$$

For $k>0$, we observe that the chosen time points $t_1, \ldots , t_k$ are prior to time *t*, hence $h_i(Z^{(b)}_{t_i})$ is $\mathcal {F}^{Z^{(b)}}_t$ measurable. By this we have that

$$\begin{aligned}&\mathbb {E}_z \left[ \left(\, f(Z^{(b)}_{t\mathop{+}s}) - f(Z^{(b)}_t) - \int _t^{t\mathop{+}s} g^{(b)}(Z^{(b)}_v) \, \mathrm{d}v \right) \prod _{i\mathop{=}1}^k h_i(Z^{(b)}_{t_i}) \right] \\&= \mathbb {E}_z \left[ \mathbb {E}_z \left[ \left(\, f(Z^{(b)}_{t\mathop{+}s}) - f(Z^{(b)}_t) - \int _t^{t\mathop{+}s} g^{(b)}(Z^{(b)}_v) \, \mathrm{d}v \right) \,\left| \, \mathcal {F}^{Z^{(b)}}_t\right. \right] \prod _{i\mathop{=}1}^k h_i(Z^{(b)}_{t_i}) \right] = 0. \end{aligned}$$

Therefore, similar to the case $k=0$ we can rewrite the difference as

$$\begin{aligned} \mathbb {E}_z \left[ \left( \int _t^{t\mathop{+}s} g^{(b)}(Z^{(b)}_v) - g (Z^{(b)}_v) \, \mathrm{d}v \right) \prod _{i\mathop{=}1}^k h_i(Z^{(b)}_{t_i}) \right] . \end{aligned}$$

The functions $h_i$ are in $C_b$, hence we can bound the absolute value of the product uniformly by some constant $\tilde{c}$ and get

$$\begin{aligned} \lim _{b \mathop{\rightarrow} \infty }&\left| \mathbb {E}_z \left[ \left( \int _t^{t\mathop{+}s} g^{(b)}(Z^{(b)}_v) - g (Z^{(b)}_v) \, \mathrm{d}v \right) \prod _{i\mathop{=}1}^k h_i(Z^{(b)}_{t_i}) \right] \right| \\ {}&\le \lim _{b \mathop{\rightarrow} \infty } \tilde{c} \int _{t}^{t\mathop{+}s} \mathbb {E}_z \left[ \left| g^{(b)}(Z^{(b)}_v) - g (Z^{(b)}_v)\right| \right] \, \mathrm{d}v =0 . \end{aligned}$$

$$\square$$

#### Corollary 6

The process $(X^{(b)}, m^{(b)}, \lambda ^{(b)})$ converges weakly against the original PDMP as $b \rightarrow \infty$.

#### Proof

This, is a direct consequence of Theorem 5 and Theorem 8.2 in (Ethier and Kurtz 2009, pp. 226–227). $\square$

### Convergence of the Discrete Processes

Now we will use the same ideas as before, but on the set

$$\begin{aligned} \mathcal {D}^{(b)} := \left\{ f \in \mathcal {D} \, \left| \, f \text { and } \delta _\phi \ f \text { are Lipschitz and } \, \mathcal {A}^{(b)}f \in C_b\right. \right\} . \end{aligned}$$

Again we define a contraction semigroup $T^{(b)}_t f(x,m,\lambda ):= \mathbb {E}_{(x,\kern.10em m,\kern.10em \lambda )} \left[\, f(X^{(b)}_t, m^{(b)}_t, \lambda ^{(b)}_t)\right]$ and want to show that this semigroup is strongly continuous at 0 over the set $\mathcal {D}^{(b)}$.

#### Lemma 7

Let *f* be in $\mathcal {D}^{(b)}$ . Then, $T^{(b)}_t f$ and $\delta _\phi T^{(b)}_t f$ are Lipschitz continuous.

#### Proof

Let $f \in \mathcal {D}^{(b)}$ be arbitrary and consider for $x \ne y$

$$\begin{aligned} \left| T^{(b)}_tf(x,m,\lambda ) -T^{(b)}_t f(y,m,\lambda )\right| =&\ \left| \mathbb {E}_{(x,\kern.10em m,\kern.10em \lambda )}\left[\, f(X^{(b)}_t,m^{(b)}_t,\lambda ^{(b)}_t)\right]\right.\\& -\left. \mathbb {E}_{(y,m,\lambda )}\left[\, f(X^{(b)}_t,m^{(b)}_t,\lambda ^{(b)}_t)\right] \right| . \end{aligned}$$

The altered initial condition in the first variable only affects the surplus process. Let $\tilde{X}^{(b)}_t$ be the reserve process with initial capital *y* and $X^{(b)}_t$ the corresponding process with starting value *x*. By the linear structure of the surplus process, we see that $\tilde{X}^{(b)}_t(\omega )= (y-x)+X^{(b)}_t(\omega )$ for all $t \ge 0$ and all $\omega \in \Omega$. By this we get that

$$\begin{aligned}&\left| \mathbb {E}_{(x,\kern.10em m,\kern.10em \lambda )}\left[\, f(X^{(b)}_t,m^{(b)}_t,\lambda ^{(b)}_t)\right] - \mathbb {E}_{(y,m,\lambda )}\left[\, f(X^{(b)}_t,m^{(b)}_t,\lambda ^{(b)}_t)\right] \right| \\&= \left| \mathbb {E}_{(x,\kern.10em m,\kern.10em \lambda )}\left[\, f(X^{(b)}_t,m^{(b)}_t,\lambda ^{(b)}_t)-f((y-x)+X^{(b)}_t,m^{(b)}_t,\lambda ^{(b)}_t)\right] \right| \\ &\le \mathbb {E}_{(x,\kern.10em m,\kern.10em \lambda )}\left[ \,\left| f(X^{(b)}_t,m^{(b)}_t,\lambda ^{(b)}_t)-f((y-x)+X^{(b)}_t,m^{(b)}_t,\lambda ^{(b)}_t)\right| \,\right] \le L \left| y-x\right| , \end{aligned}$$

where *L* denotes the Lipschitz constant of *f* with respect to $\Vert .\Vert _1$. The same idea leads to a preserved Lipschitz-continuity in the second variable.

Now we want to show that this holds for the third variable too. Here, things get a little more complicated, since small changes in the intensity process influence all three processes. Let us now consider a realization $\lambda ^{(b)}_t$ of the intensity process with initial condition $\lambda ^{(b)}_0\mathop{=}\lambda$ and for some $h>0$ the altered realization $\tilde{\lambda }^{(b)}$ with starting value $\lambda + h$ and take a look at the difference of those processes. If no shock event appeared until time *t*, or shocks happened but $\tilde{\lambda }^{(b)}$ did not hit $\lambda _{max}(b)$, the relation between those processes is $\tilde{\lambda }^{(b)}_t = \lambda ^{(b)}_t + h e^{-\gamma t}$. Otherwise, the difference decreases and may even become 0 if both, $\lambda ^{(b)}$ and $\tilde{\lambda }^{(b)}$ hit $\lambda _{max}(b)$.

As already mentioned, the difference in the starting intensity leads to a change in the surplus process too. To be precise, we again consider two realizations, $\tilde{X}^{(b)}_t$ with starting intensity $\lambda +h$ and $X^{(b)}_t$ corresponding to $\lambda ^{(b)}_0\mathop{=}\lambda$. They are related by $\tilde{X}^{(b)}_t = X^{(b)}_t - \sum _{i\mathop{=}1}^{\tilde{N}_t} \tilde{U}_i$, where $\tilde{N}$ is a counting process with intensity $\tilde{\lambda }^{(b)}_t - \lambda ^{(b)}_t \le h e^{- \gamma t}$ and additional i.i.d. claims $\tilde{U}_i \sim U$ independent of all $U_i$.

Finally, the corresponding realizations $\tilde{m}^{(b)}_t$ and $m^{(b)}_t$ may relate in three different ways. The first case is that $N^{(b)}_t >0$ and the last jump before time *t* is due to $\tilde{N}$. In this case $m^{(b)}_t$ and $\tilde{m}^{(b)}_t$ are not equal but i.i.d. random variables. In the second case, $N^{(b)}_t=0$ but $\tilde{N}_t$ is not. In this case $m^{(b)}_t=m$ and $\tilde{m}^{(b)}_t \sim \tilde{U}$. In the remaining case we have that $\tilde{m}^{(b)}_t = m^{(b)}_t$.

Having this in mind we now consider the following:

$$\begin{aligned} \left| T^{(b)}_t f(x,m,\lambda +h) - T^{(b)}_t f(x,m,\lambda ) \right|=&\ \left| \mathbb {E}_{(x,\kern.10em m,\kern.10em \lambda )} \left[\, f(\tilde{X}^{(b)}_t, \tilde{m}^{(b)}_t, \tilde{\lambda }^{(b)}_t) - f(X^{(b)}_t,m^{(b)}_t,\lambda ^{(b)}_t) \right] \right| \\ \le&\ \mathbb {E}_{(x,\kern.10em m,\kern.10em \lambda )} \left[ \left|\, f(\tilde{X}^{(b)}_t , \tilde{m}^{(b)}_t , \tilde{\lambda }^{(b)}_t) - f(X^{(b)}_t, \tilde{m}^{(b)}_t, \tilde{\lambda }^{(b)}_t) \right| \right] \\ {}&+\left| \mathbb {E}_{(x,\kern.10em m,\kern.10em \lambda )} \left[\, f( X^{(b)}_t , \tilde{m}^{(b)}_t , \tilde{\lambda }^{(b)}_t) - f(X^{(b)}_t, m^{(b)}_t, \tilde{\lambda }^{(b)}_t) \right] \right| \\ {}&+\mathbb {E}_{(x,\kern.10em m,\kern.10em \lambda )} \left[ \left|\, f( X^{(b)}_t , m^{(b)}_t , \tilde{\lambda }^{(b)}_t) - f(X^{(b)}_t, m^{(b)}_t, \lambda ^{(b)}_t) \right| \right] . \end{aligned}$$

Since *f* is Lipschitz, the third term can be bounded by

$$\begin{aligned} \mathbb {E}_{(x,\kern.10em m,\kern.10em \lambda )} \left[ \left|\, f( X^{(b)}_t , m^{(b)}_t , \tilde{\lambda }^{(b)}_t) - f(X^{(b)}_t, m^{(b)}_t, \lambda ^{(b)}_t) \right| \right] \le L \mathbb {E}_{(x,\kern.10em m,\kern.10em \lambda )} \,\left[ \left| \tilde{\lambda }^{(b)}_t-\lambda ^{(b)}_t \right| \right] \le L h e^{-\gamma t} . \end{aligned}$$

By the same arguments, we can bound the first term by

$$\begin{aligned} \mathbb {E}_{(x,\kern.10em m,\kern.10em \lambda )} \left[ \left|\, f(\tilde{X}^{(b)}_t , \tilde{m}^{(b)}_t , \tilde{\lambda }^{(b)}_t) - f(X^{(b)}_t, \tilde{m}^{(b)}_t, \tilde{\lambda }^{(b)}_t) \right| \right] \le L \mathbb {E}_{(x,\kern.10em m,\kern.10em \lambda )} \,\left[ \left| \sum _{i\mathop{=}1}^{\tilde{N}_t} \tilde{U}_i \right| \right] \le \frac{L}{\gamma } \mathbb {E}\left[ U\right]\, h . \end{aligned}$$

The second term can be reduced to

$$\begin{aligned}&\left| \mathbb {E}_{(x,\kern.10em m,\kern.10em \lambda )} \left[\, f( X^{(b)}_t , \tilde{m}^{(b)}_t , \tilde{\lambda }^{(b)}_t) - f(X^{(b)}_t, m^{(b)}_t, \tilde{\lambda }^{(b)}_t) \right] \right|\\ & = \left| \mathbb {E}_{(x,\kern.10em m,\kern.10em \lambda )} \left[ \left(\, f( X^{(b)}_t , \tilde{U} , \tilde{\lambda }^{(b)}_t) - f(X^{(b)}_t, m, \tilde{\lambda }^{(b)}_t) \right) I_{\left\{ N^{(b)}_t \,=\,0 \right\} } I_{\left\{ \tilde{N}_t\,>\,0 \right\} }\right] \right| \\ &\le 2\Vert f\Vert _{\infty } \mathbb {P}_{\lambda } \left[ \tilde{N}_t >0 \right] \le 2 \Vert f\Vert _{\infty }h \frac{1-\exp \left( - \frac{1-e^{-\gamma t}}{\gamma }h \right) }{h} \le 2 \Vert f\Vert _{\infty }h \frac{1-e^{-\gamma t}}{\gamma }. \end{aligned}$$

Using these results, we get that there exists a constant *K* such that

$$\begin{aligned} \left| T^{(b)}_t f(x,m,\lambda +h) - T^{(b)}_t f(x,m,\lambda ) \right| \le K h , \end{aligned}$$

for all positive $\lambda$ and *h*. Consequently, $T^{(b)}_tf$ is Lipschitz for all $f \in \mathcal {D}^{(b)}$.

To show the Lipschitz continuity of the path-derivative $\delta _\phi T^{(b)}_t f$, we use the following representation derived in the proof of Theorem 7.7.4 in Jacobsen (2006):

$$\begin{aligned} \delta _\phi T^{(b)}_t f (x,m,\lambda ) =&\ T^{(b)}_t \left( \mathcal {A}^{(b)} f\right) (x,m,\lambda ) \\ {}&+ \lambda \int _{(0,U_{max}(b)]} T^{(b)}_tf(x,m,\lambda )-T^{(b)}_t f(x-u,u,\lambda ) \, F_{U^{(b)}} ( \mathrm{d}u) \\ {}&+ \rho \int _{(0,Y_{max}(b)]} T^{(b)}_tf(x,m,\lambda ) - T^{(b)}_tf(x,m,\lambda +y) \, F_{Y^{(b)}}(\mathrm{d}y). \end{aligned}$$

Since $T^{(b)}_t$ preserves Lipschitz continuity, we know that the integral terms are indeed Lipschitz. Now we just have to show that for every $f \in \mathcal {D}^{(b)}$, the function $\mathcal {A}^{(b)} f$ is Lipschitz too. Let $z_1 := (x_1,m_1,\lambda _1)$ and $z_2 := (x_2,m_2,\lambda _2)$ two suitable points and consider

$$\begin{aligned} \left| \mathcal {A}^{(b)} f(z_1) - \mathcal {A}^{(b)} f(z_2)\right| =&\ \left| \delta _\phi \ f(z_1) - \delta _\phi \ f(z_2) + \lambda _1 \int _{(0,U_{max}(b)]} f(x_1-u,u,\lambda _1) \, F_{U^{(b)}} ( \mathrm{d}u) \right. \\ {}&- \lambda _2 \int _{(0,U_{max}(b)]} f(x_2-u,u,\lambda _2) \, F_{U^{(b)}}(\mathrm{d}u) \\ {}&- \rho (\,f(z_1)-f(z_2)) - \lambda _1 f(z_1) + \lambda _2 f(z_2) \\ {}&\left. + \rho \int _{(0,Y_{max}(b)]} f(x_1,m_1,\lambda _1+y) - f(x_2, m_2,\lambda _2+y) \, F_{Y^{(b)}} ( \mathrm{d}y) \right| \end{aligned}$$

Using the triangle inequality and the Lipschitz continuity of $\delta _\phi \ f$ and *f* we get that there is a constant *K* such that the above is less or equal to

$$\begin{aligned}&K \Vert z_1-z_2 \Vert _1 + \lambda _1 \left| \int _0^\infty f(x_1-u,u,\lambda _1) -f(x_2-u,u,\lambda _2) \, F_{U^{(b)}} ( \mathrm{d}u) \right| \\ {}&+ \left| ( \lambda _1-\lambda _2) \int _0^\infty f(x_2-u,u,\lambda _2) \, F_{U^{(b)}} ( \mathrm{d}u) \right| + \lambda _1 \left|\, f(z_1)-f(z_2) \right| + \left| (\lambda _1-\lambda _2)\, f(z_2) \right| . \end{aligned}$$

For all *u*, the Lipschitz continuity of *f* gives us the existence of positive constants *L* and $\tilde{L}$ such that

$$\begin{aligned} \left|\, f(x_1-u, u, \lambda _1) - f(x_2-u,u,\lambda _2)\right| \le L \max \left\{ \left| x_1-x_2\right| ,\left| \lambda _1-\lambda _2\right| \right\}&\le L \Vert z_1 - z_2 \Vert _{\infty } \\ {}&\le \tilde{L} \Vert z_1-z_2\Vert _{1}, \end{aligned}$$

where the last inequality is given by the equivalence of norms in finite dimensional spaces. Further, we get that there is a constant $\tilde{c}$ with

$$\begin{aligned} \left| (\lambda _1-\lambda _2)\, f(z_2) \right| \le \Vert f\Vert _{\infty } \Vert z_1 -z_2\Vert _{\infty } \le \tilde{c} \Vert z_1-z_2\Vert _{1}. \end{aligned}$$

Using these inequalities and the boundedness of $\lambda ^{(b)}$ by $\lambda _{max}(b)$ we get that

$$\begin{aligned} \left| \mathcal {A}^{(b)} f(z_1) - \mathcal {A}^{(b)} f(z_2)\right| \le K \Vert z_1-z_2 \Vert _1 + 2\tilde{L} \Vert z_1-z_2 \Vert _1 + 2 \tilde{c} \Vert z_1-z_2\Vert _{1}. \end{aligned}$$

By this, the function $\mathcal {A}^{(b)}f$ is Lipschitz continuous and further, the same holds for $\delta _\phi T^{(b)}_t f$. $\square$

#### Lemma 8

The family $\left\{ T^{(b)}_t \right\} _{ t \,\ge\, 0}$ is a strongly continuous contraction semigroup on $\mathcal {D}^{(b)}$.

#### Proof

By the results shown in Lemma 7 and the ideas of the proof of Lemma 4, we get that $T^{(b)}_t$ maps $\mathcal {D}^{(b)}$ into itself and by the boundedness of $\mathcal {A}^{(b)}f$ we get the strong continuity property. $\square$

#### Lemma 9

Let $f \in \mathcal {D}^{(b)}$ be arbitrary. Then, there exists a positive constant $\tilde{K}$ such that for every point $(x_i,x_l,\lambda _j)$ in the state space of the bounded and discrete process

$$\begin{aligned} \left| \mathcal {A}^{(h,\kern.10em b)} f(x_i,x_l,\lambda _j) - \mathcal {A}\,f(x_i,x_l,\lambda _j)\right| \le \tilde{K} h . \end{aligned}$$

#### Proof

Let $f \in \mathcal {D}^{(b)}$ be arbitrary and $(x_i,x_l,\lambda _j)$ a point in the state space of our bounded and discrete process. If we consider the difference between the two generators we get by the triangle inequality that

$$\begin{aligned}&\left| \mathcal {A}^{(h,\kern.10em b)} f(x_i,x_l,\lambda _j) \right. -\left. \mathcal {A}^{(b)} f(x_i,x_l,\lambda _j) \right| \\&\le \left| \frac{f(x_i +ch, x_l, \lambda _j e^{-\gamma h}) - f(x_i,x_l,\lambda _j) }{h} - \delta _\phi \ f(x_i,x_l,\lambda _j) \right| \\ {}&\quad + \lambda _j \left| \sum _{k\mathop{=}1}^{N_U} f(x_i\mathop{-}x_k, x_k, \lambda _j) p^U_k - \int _{(0,U_{max}(b)]} f(x_i-u,u,\lambda _j) \, F_{U^{(b)}}(\mathrm{d}u) \right| \\ {}&\quad + \rho \left| \sum _{k\mathop{=}1}^{N_Y(j)} f(x_i,x_l,\lambda _{j\mathop{+}k}) p^Y_k(j) - \int _{(0,Y_{max}(b)]} f(x_i,m, \lambda _j+y) \, F_{Y^{(b)}} (\mathrm{d}y) \right| . \end{aligned}$$

Let us first consider the second term. We can rewrite the difference as

$$\begin{aligned}&\lambda _j \left| \sum _{k\mathop{=}1}^{N_U} f(x_i\mathop{-}x_k, x_k, \lambda _j) p^U_k - \int _{(0,U_{max}(b)]} f(x_i-u,u,\lambda _j) \, F_{U^{(b)}}(\mathrm{d}u) \right| \\ {}&= \lambda _j \left| \sum _{k\mathop{=}1}^{N_U} \int _{(x_{k\mathop{-}1}, x_k]} f(x_i\mathop{-}x_k,x_k,\lambda _j) - f(x_i-u,u,\lambda _j) \, F_{U^{(b)}}(\mathrm{d}y) \right| . \end{aligned}$$

By the Lipschitz continuity of *f* and the boundedness of $\lambda _j$, we get that this is less or equal to $2 L \lambda _{max}(b)ch,$ where *L* denotes a Lipschitz constant of *f*. By the same idea, we can bound the third term by

$$\begin{aligned} L \rho \lambda _{max}(b)(1-e^{-\gamma h}) \le L\rho \lambda _{max} (b) \gamma h. \end{aligned}$$

For the second term we define the function $g: [0,\infty ) \rightarrow \mathbb {R}$ by

$$\begin{aligned} g(t) = f(x_i+ct, x_l, \lambda _je^{-\gamma t}). \end{aligned}$$

This is a Lipschitz continuous function in one real variable. Hence, it is differentiable almost everywhere and at every *u*, where *g* is differentiable the equality $g'(u) = \delta _\phi \ f(x+cu,x_l,\lambda e^{-\gamma u})$ holds. By this we get that

$$\begin{aligned}\left| \frac{f(x_i+ch,x_l,\lambda _j e^{-\gamma h})-f(x_i,x_l,\lambda _j)}{h}- \delta _\phi \ f(x_i,x_l,\lambda ) \right|& = \frac{1}{h}\left| \int _0^h g'(u) - \delta _\phi \ f(x_i,x_l,\lambda _j) \, \mathrm{d}u \right| \\ {}&= \frac{1}{h}\left| \int _0^h \delta _\phi \ f(x_i+cu,x_l,\lambda _j e^{-\delta u}) - \delta _\phi \ f(x_i,x_l,\lambda _j) \, \mathrm{d}u \right| \\ {}&\le \tilde{L} (ch+\lambda _{max}(b) (1-e^{-\gamma h})) \le \tilde{L} (c+ \lambda _{max}(b) \gamma ) h, \end{aligned}$$

where $\tilde{L}$ is a Lipschitz constant of $\delta _\phi \ f$. By this we get that

$$\left| \mathcal {A}^{(h,\kern.10em b)} f(x_i,x_l,\lambda _j) - \mathcal {A}^{(b)} f(x_i,x_l,\lambda _j) \right| \le ( \tilde{L} (c+ \gamma \lambda _{max} (b))+ 2 L \lambda _{max}(b)c +L\rho \lambda _{max} (b) \gamma )h .$$

$$\square$$

Equivalently to Theorem 5, we prove the following lemma.

#### Lemma 10

Let $f \in \mathcal {D}^{(b)}$ be arbitrary but fixed. Then, for all $t,s >0$, $k\ge 0$, $h_1, \ldots ,h_k \in C_b$ and $t_1<t_2< \ldots < t_k \le t$ we have that

$$\begin{aligned} \mathbb {E}_{(x_i,m,\lambda _j)} \left[ \left( \int _t^{t\mathop{+}s} \left( \mathcal {A}^{(h,\kern.10em b)} f(X^{(h,\kern.10em b)}_v, m^{(h,\kern.10em b)}_v,\lambda ^{(h,\kern.10em b)}_v)\right.\right.\right.& -\left.\left.\left. \mathcal {A}^{(b)} f(X^{(h,\kern.10em b)}_v, m^{(h,\kern.10em b)}_v,\lambda ^{(h,\kern.10em b)}_v) \right) \, \mathrm{d}v \right) \right. \\&\times \left. \prod _{l=1}^k h_l\left( X^{(h,\kern.10em b)}_{t_l}, m^{(h,\kern.10em b)}_{t_l}, \lambda ^{(h,\kern.10em b)}_{t_l} \right) \right] \rightarrow 0 , \end{aligned}$$

as $$h \rightarrow 0$$.

#### Proof

The functions $h_l$ are bounded, hence there is a constant *L* such that

$$\begin{aligned}& \left| \left( \int _t^{t\mathop{+}s} \left( \mathcal {A}^{(h,\kern.10em b)} f(X^{(h,\kern.10em b)}_v, m^{(h,\kern.10em b)}_v,\lambda ^{(h,\kern.10em b)}_v) -\mathcal {A}^{(b)} f(X^{(h,\kern.10em b)}_v, m^{(h,\kern.10em b)}_v,\lambda ^{(h,\kern.10em b)}_v) \right) \, \mathrm{d}v \right) \right. \\&\quad\cdot \left. \prod _{l=1}^k h_l\left( X^{(h,\kern.10em b)}_{t_l}, m^{(h,\kern.10em b)}_{t_l}, \lambda ^{(h,\kern.10em b)}_{t_l} \right) \right| \\& \le L \int _t^{t\mathop{+}s} \left| \mathcal {A}^{(h,\kern.10em b)} f(X^{(h,\kern.10em b)}_v, m^{(h,\kern.10em b)}_v,\lambda ^{(h,\kern.10em b)}_v) - \mathcal {A}^{(b)} f(X^{(h,\kern.10em b)}_v, m^{(h,\kern.10em b)}_v,\lambda ^{(h,\kern.10em b)}_v) \right| \, \mathrm{d}v. \end{aligned}$$

By the boundedness derived in Lemma 9, we get that there is a constant $\tilde{K}$ such that

$$\begin{aligned} \mathbb {E}_{(x_i,m,\lambda _j)} \left[ \int _t^{t\mathop{+}s} \left| \mathcal {A}^{(h,\kern.10em b)} f(X^{(h,\kern.10em b)}_v, m^{(h,\kern.10em b)}_v,\lambda ^{(h,\kern.10em b)}_v) - \mathcal {A}^{(b)} f(X^{(h,\kern.10em b)}_v, m^{(h,\kern.10em b)}_v,\lambda ^{(h,\kern.10em b)}_v) \right| \, \mathrm{d}v \right] \le \tilde{K} h s, \end{aligned}$$

which tends to 0 as $h \rightarrow 0$. $\square$

#### Theorem 11

The process $(X^{(h,\kern.10em b)}, m^{(h,\kern.10em b)}, \lambda ^{(h,\kern.10em b)})$ converges in distribution to the bounded process $(X^{(b)}, m^{(b)}, \lambda ^{(b)})$ as $h \rightarrow 0$.

#### Proof

We obtain this by the result of Lemma 10 and Theorem 8.2 in (Ethier and Kurtz 2009, p. 226). $\square$

#### Theorem 12

The Markov chain with finite state space converges weakly against the discrete process.

#### Proof

This can be proven as the weak convergence of the other processes using the convergence of the generators on the set

$$\begin{aligned} \mathcal {D}^{(h,\kern.10em b)} = \left\{ f \in C_b \, \left| \, \mathcal {A}^{(h,\kern.10em b)} f \in C_b, \, \lim _{x \rightarrow \infty } f(x,m,\lambda ) =0 \, \text { uniformly in } m \text { and } \lambda \right. \right\} , \end{aligned}$$

where $\mathcal {A}^{(h,\kern.10em b)}$ generates a strongly continuous contraction semigroup. $\square$

### Convergence of the Gerber-Shiu Functions

#### Theorem 13

Let $g_\kappa$ be an arbitrary Gerber-Shiu function and $g^{(\bar{x},h,b)}_\kappa$ the corresponding GS-function of the Markov chain with finite state space. For $(x,\lambda )$ let $j=N_\lambda - \left\lfloor \frac{\ln \left( \lambda _{max}\right) -\ln \left( \lambda \right) }{h\gamma }\right\rfloor$, and $i = \left\lfloor \frac{x}{hc}\right\rfloor$. Then, we have

$$\begin{aligned} \lim _{b \mathop{\rightarrow} \infty } \lim _{h \mathop{\rightarrow} 0} \lim _{\bar{x} \rightarrow \infty } \left| g^{(\bar{x},h,b)}_\kappa (x_i, \lambda _j) -g_\kappa (x,\lambda ) \right| =0. \end{aligned}$$

#### Proof

By the proof of Lemma 5.14 in Kritzer et al. (2019), we have that our GS-function is a Skorokhod-continuous function of the process. By the weak convergence of the underlying processes, we know that the penalty functions converge too. Consequently,

$$\begin{aligned} \lim _{b \mathop{\rightarrow} \infty } \lim _{h \mathop{\rightarrow} 0} \lim _{\bar{x} \,\rightarrow \,\infty } \left| g^{(\bar{x},h,b)}_\kappa (x_i, \lambda _j) -g_\kappa (x,\lambda ) \right| =0. \end{aligned}$$

$$\square$$

## Examples

In this section, we give some explicit examples of Gerber-Shiu functions and corresponding numerical approximations in a Markovian shot-noise model with the following specific parameters. We choose decay parameter $\gamma =1$, intensity of the underlying Poisson process $\rho =1.5$ and premium rate $c= \frac{15}{4}$. Further, we assume that the shock events $Y_i$ and the claim events $U_i$ are exponentially distributed with mean 1. All computations and simulations are made on a standard notebook with an Intel Core i5.10210U processor at 1.60 GHz and 16 GB of RAM.

### Laplace-Transform Function of the Time of Ruin

**Fig. 1:**
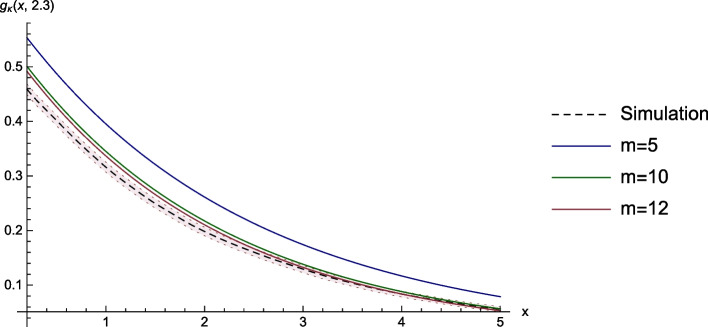
Laplace transform of the time of ruin and the corresponding numerical approximations

The first example is the GS-function $g_\kappa ^{(1)}:=\mathbb {E}_{(x,\kern.10em\lambda )}\left[ e^{-\kappa \tau }I_{\left\{ \tau \mathop{<} \infty \right\} }\right] ,$ i.e. the Laplace transform of $\tau$, with $\kappa = 0.1,$ and for fixed $\lambda =2.3$. In Fig. 1, the function in black is a Monte Carlo simulation using 10000 sample paths and the red area is the corresponding 95-percent confidence interval. For the numerical approximations, we choose $\bar{x}=50$, $\lambda _{max}=4.5$, $U_{max}=10$, $Y_{max}=4.5$ and $h=\frac{1}{cm}$ for $m \in \left\{ 5,10,12\right\}$.

As we can see in Table 1, the main advantage of the numerical method is the speed of computation. The scheme with $h\approx 0.02$ needed about 38 minutes whereas the computation of the corresponding simulation needed approximately 27 hours.

**Table 1:** Computation time of the surface and errors of the approximations at the point $x=3$ and $\lambda =2.3$

m	Minutes	Value	Abs. error	Rel. error
5	0.46	0.174	0.045	0.347
10	17	0.138	0.009	0.068
12	38	0.133	0.003	0.026
Sim.	1619	0.129	-	-

### Discounted Surplus Before Ruin

**Fig. 2:**
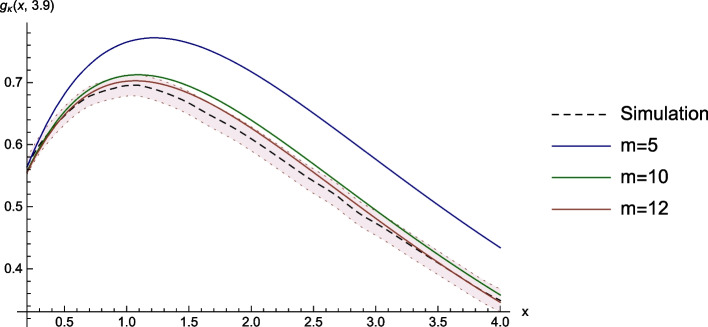
Discounted surplus before ruin and numerical approximations

Here, we consider the same setting as in the previous example, but now with penalty function $g^{(2)}_\kappa (x,\lambda ) = \mathbb {E}_{(x,\kern.10em\lambda )}\left[ e^{-\kappa \tau } X_{\tau -} I_{\left\{ \tau \mathop{<} \infty \right\} }\right]$. Again, the black function in Fig. 2 is a MC simulation from 10000 paths, which we will use as a reference solution. This time, the plot shows the behaviour of the GS-function in *x* for fixed $\lambda = 3.9$. As before, the numerical approximations are calculated with parameter $\bar{x}=50$, $\lambda _{max}=4.5$, $U_{max}=10$, $Y_{max}=4.5$ and $h=\frac{1}{cm}$ for $m \in \left\{ 5,10,12\right\}$.

The function $w(x,y)=x$ is continuous but not bounded. We bypass this problem by considering penalty functions of the form $\tilde{w}(x,y) = \min (x,n)$ for $n \in \mathbb {N}$. These functions are continuous and bounded; hence, the theory derived before is applicable. Further, the sequence $\left\{ e^{-\kappa \tau } \min \left( X_{\tau -},n\right) I_{\left\{ \tau \mathop{<} \infty \right\} } \right\} _{n\, \in\, \mathbb {N}}$ is monotone increasing. Consequently, the approximations converge for $n \rightarrow \infty$ by monotone convergence. As we can see in Table 2, the behaviour in terms of computing time and relative error is similar to the corresponding values in the example of the Laplace transform.

**Table 2:** Computation time of the surface, and errors of the approximations at the point $x=2.5$ and $\lambda =3.9$

m	Minutes	Value	Abs. error	Rel. error
5	0.45	0.65	0.11	0.203
10	15	0.57	0.03	0.052
12	38	0.55	0.01	0.027
Sim.	1622	0.54	-	-

### Ruin Probability

**Fig. 3:**
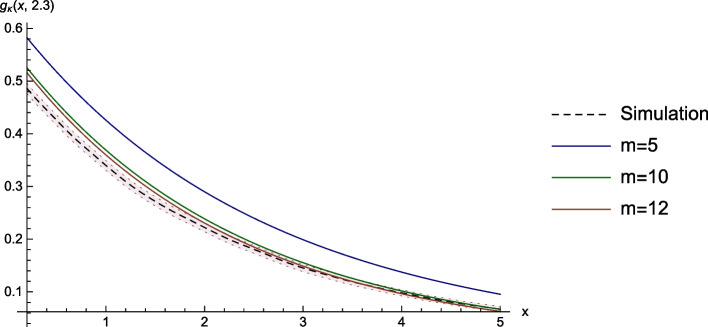
Ruin probability and numerical approximations

As a third example, we consider the ruin probability $g^{(3)}_\kappa (x,\lambda ) = \mathbb {E}_{(x,\kern.10em\lambda )}\left[ I_{\left\{ \tau \mathop{<} \infty \right\} }\right]$. The reference solution is again obtained by MC-simulation, and the bounds $\bar{x}$, $\lambda _{max}$, $U_{max}$, and $Y_{max}$ are chosen as in the previous examples. An illustration of the simulation and the numerical approximations for fixed $\lambda =2.3$ can be seen in Fig. 3.

In Table 3, we see that the run time and the relative error are very similar to the first two examples. Again, even in the finest step size considered, the numerical scheme beats the simulation by a factor of $\approx 40$ in terms of computation time, which is the main advantage of our approach.

**Table 3:** Computation time of the surface and errors of the approximations at the point $x=2.5$ and $\lambda =2.3$

m	Minutes	Value	Abs. error	Rel. error
5	0.46	0.240	0.059	0.327
10	15	0.192	0.011	0.063
12	38	0.185	0.004	0.023
Sim.	1614	0.181	-	-

### Empirical Convergence Order

Another topic of interest is the convergence order of numerical schemes. This has been studied for example by Chau et al. (2015), who considered GS-functions in a Lévy subordinator model. Numerical methods to solve integro-differential equations related to ours are also derived in Brunner (1988), who proposed spline collocation methods for ordinary Volterra integro-differential equations. He was able able achieve a convergence order up to order 2*m*, given that the coefficients of the Volterra equation are 2*m* times continuous differentiable.

Since we consider partial integro-differential equations, we cannot use his results to obtain a theoretical convergence order. Alternatively, we compute the empirical order of convergence of our numerical scheme in the examples given before. For this, we consider two different approaches. The first is the estimated order of convergence (EOC) as defined in (Steinbach 2008, p. 253). For a sequence $\left\{ x_n\right\} _{n\, \ge\, 0}$ with limit *x*, we define the sequence of absolute errors by $e_n := \left| x-x_n\right|$. Assuming that $e_n \approx C n^{-\rho }$ for some fixed constant, i.e. that the sequence converges with order $\rho$, we divide by $e_{n\,-\,1}$ and get $\frac{e_n}{e_{n\,-\,1}} \approx (\frac{n}{n\,-\,1})^{-\rho }$. Applying the logarithm and dividing by $\ln (\frac{n}{n\,-\,1})$ gives us the EOC

$$\hat{\rho }_n = \frac{\ln \left( \frac{e_n}{e_{n\,-\,1}}\right) }{\ln \left( \frac{n\,-\,1}{n}\right) }.$$

The second procedure uses the same assumption $e_n \approx C n^{-\rho }$, or equivalently $\ln (e_n) \approx \ln (C) - \rho \ln (n)$. Having this form, we use a linear regression approach to get an estimator $\tilde{\rho }$ for the parameter $\rho$, as it is used by Chau et al. (2015).

We are interested in the convergence behaviour of the sequence of our numerical approximations at some fixed points $(x_i,\lambda _j)$ as the fineness of the discretization tends to 0. To determine the error terms $e_n$ correctly, we have to know the limit of this sequence, which is not the GS-function of our original process, but the GS-function of the bounded process, which we obtain by simulation.

In the following examples, we fix the bounds $\bar{x}=50$, $Y_{max}=4.5$, $U_{max}=10$ and $\lambda _{max}=4.5$ and the GS-functions $g_\kappa ^{(1)}$, $g_\kappa ^{(2)}$, and $g_\kappa ^{(3)}$ as before. Then, we consider the sequence of numerical approximations with step size $h= \frac{1}{cm}$ for $m \in \left\{ 1, \ldots , 12\right\}$ and compute the EOC and the regression estimate $\tilde{\rho }$ at the points (0.4, 2.3), (1.4, 2.3), and (2.5, 2.3). As we can see in Table 4, it seems plausible that we observe a linear convergence behaviour.

**Table 4:** Table of EOC and estimates done by regression approach

	Laplace transform			Surplus before ruin			Ruin probability		
m	$x=0.4$	$x=1.4$	$x=2.5$	$x=0.4$	$x=1.4$	$x=2.5$	$x=0.4$	$x=1.4$	$x=2.5$
2	0.739	0.924	1.093	1.159	0.955	1.130	0.801	1.014	1.208
3	0.895	1.047	1.186	0.872	1.034	1.166	0.965	1.125	1.263
4	0.929	1.056	1.172	0.821	1.038	1.142	1.013	1.139	1.240
5	0.932	1.049	1.149	0.823	1.029	1.124	1.035	1.143	1.218
6	0.924	1.036	1.131	0.861	1.028	1.107	1.046	1.145	1.206
7	0.915	1.026	1.115	0.835	1.016	1.097	1.057	1.151	1.197
8	0.904	1.015	1.104	0.837	1.010	1.086	1.064	1.157	1.195
9	0.892	1.005	1.094	0.827	1.003	1.080	1.073	1.165	1.194
10	0.881	0.997	1.087	0.810	0.996	1.073	1.081	1.175	1.197
11	0.869	0.988	1.080	0.814	0.993	1.070	1.087	1.185	1.201
12	0.858	0.981	1.075	0.793	0.985	1.065	1.096	1.197	1.207
$\tilde{\rho }$	0.911	1.032	1.136	0.836	1.022	1.114	1.037	1.148	1.218

**Fig. 4:**
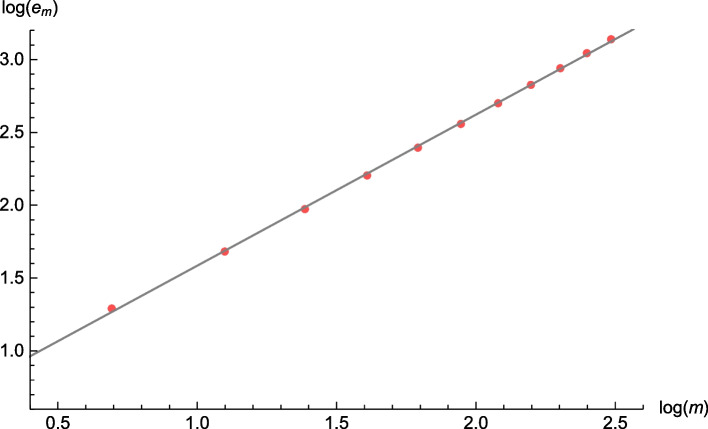
Linear regression line with slope $\tilde{\rho }= 1.037$ and observed errors

In Fig. 4 we see the linear function obtained by regression of the approach $-\ln (e_n) = \ln (C) + \rho \ln (n)$ for the ruin probability in the point (0.4, 2.3) and the corresponding observed values in red. The coefficient of determination $R^2 = 0.9996$ indicates that there is indeed a linear relationship between $\ln (e_n)$ and $\ln (n)$, i.e. that the assumption $e_n \approx C n^{-\rho }$ is reasonable, and that $\tilde{\rho }\approx 1.037$ is a good estimation of the true convergence order in this example.

## Data Availability

The datasets generated during and/or analysed during the current study are available from the corresponding author on reasonable request.
